# Controversies about extended-spectrum and AmpC beta-lactamases.

**DOI:** 10.3201/eid0702.010238

**Published:** 2001

**Authors:** K S Thomson

**Affiliations:** Creighton University School of Medicine, Omaha, Nebraska 68178, USA. kstaac@creighton.edu

## Abstract

Many clinical laboratories have problems detecting extended-spectrum beta-lactamases (ESBLs) and plasmid-mediated AmpC beta-lactamases. Confusion exists about the importance of these resistance mechanisms, optimal test methods, and appropriate reporting conventions. Failure to detect these enzymes has contributed to their uncontrolled spread and sometimes to therapeutic failures. Although National Committee for Clinical Laboratory Standards recommendations exist for detecting ESBL- producing isolates of Escherichia coli and Klebsiella spp., no recommendations exist for detecting ESBLs in other organisms or for detecting plasmid-mediated AmpC beta-lactamases in any organisms. Clinical laboratories need to have adequate funding, equipment, and expertise to provide a rapid and clinically relevant antibiotic testing service in centers where these resistance mechanisms are encountered.


### Controversies about Extended-Spectrum and AmpC Beta-Lactamases

**Kenneth S. Thomson**
Creighton University School of Medicine, Omaha, Nebraska, USA

Many clinical laboratories have problems detecting extended-spectrum beta-lactamases (ESBLs) and plasmid-mediated AmpC beta-lactamases. Confusion exists about the importance of these resistance mechanisms, optimal test methods, and appropriate reporting conventions. Failure to detect these enzymes has contributed to their uncontrolled spread and sometimes to therapeutic failures. Although National Committee for Clinical Laboratory Standards recommendations exist for detecting ESBL-producing isolates of *Escherichia coli* and *Klebsiella* spp., no recommendations exist for detecting ESBLs in other organisms or for detecting plasmid-mediated AmpC beta-lactamases in any organisms. Clinical laboratories need to have adequate funding, equipment, and expertise to provide a rapid and clinically relevant antibiotic testing service in centers where these resistance mechanisms are encountered.

Extended-spectrum beta-lactamases (ESBLs) were first reported in 1983 (1), and plasmid-mediated AmpC beta-lactamases were reported in 1988 (2). Typically, ESBLs are mutant, plasmid-mediated beta-lactamases derived from older, broad-spectrum beta-lactamases (e.g., TEM-1, TEM-2, SHV-1), which have an extended substrate profile that permits hydrolysis of all cephalosporins, penicillins, and aztreonam. These enzymes are most commonly produced by *Klebsiella* spp. and *Escherichia coli* but may also occur in other gram-negative bacteria, including *Enterobacter, Salmonella, Proteus,* and *Citrobacter* spp., *Morganella morganii*, *Serratia marcescens*, *Shigella dysenteriae*, *Pseudomonas aeruginosa*, *Burkholderia cepacia*, and *Capnocytophaga ochracea* (3-9). Plasmid-mediated AmpC beta-lactamases have arisen through the transfer of chromosomal genes for the inducible AmpC beta-lactamase onto plasmids. This transfer has resulted in plasmid-mediated AmpC beta-lactamases in isolates of *E. coli, Klebsiella pneumoniae, Salmonella* spp., *Citrobacter freundii, Enterobacter aerogenes,* and *Proteus mirabilis* (10-12). To date, all plasmid-mediated AmpC beta-lactamases have similar substrate profiles to the parental enzymes from which they appear to be derived. With one exception (13), plasmid-mediated AmpCs differ from chromosomal AmpCs in being uninducible. Both ESBLs and plasmid-mediated AmpC beta-lactamases are typically associated with broad multidrug resistance (usually a consequence of genes for other antibiotic resistance mechanisms residing on the same plasmids as the ESBL and AmpC genes). A serious challenge facing clinical laboratories is that clinically relevant ESBL-mediated resistance is not always detectable in routine susceptibility tests.

Many clinical laboratories (as well as the wider medical community) are not fully aware of the importance of ESBLs and plasmid-mediated AmpCs and how to detect them; laboratories may also lack the resources to curb the spread of these resistance mechanisms (14-16). This lack of understanding or resources is responsible for a continuing failure to respond appropriately to prevent the rapid worldwide dissemination of pathogens possessing these beta-lactamases. The consequence has been avoidable therapeutic failures (sometimes fatal) in patients who received inappropriate antibiotics (17-22) and outbreaks of multidrug-resistant, gram-negative pathogens that required expensive control efforts (23).

I describe gaps in the capabilities of clinical laboratories to accurately detect and report ESBLs and plasmid-mediated AmpC beta-lactamases; discuss some of the technical difficulties involved in designing tests to detect ESBLs in organisms other than *E. coli* and *Klebsiella* spp.; correlate laboratory problems with the recent emphasis on medical cost-cutting at a time when bacterial pathogens are increasing in complexity; and propose a way to improve laboratory performance to meet the challenge of antibiotic resistance.

### Laboratory Testing for ESBLs and Plasmid-Mediated AmpC beta-Lactamases

The National Committee for Clinical Laboratory Standards (NCCLS) has issued recommendations for ESBL screening and confirmation for isolates of *E. coli* and *Klebsiella* spp., and reporting confirmed organisms (24). Compliance varies widely. Many laboratories have difficulty detecting ESBL- or AmpC-mediated resistance and may be unaware of the relevant NCCLS reporting guidelines (14). No NCCLS recommendations exist for ESBL detection and reporting for other organisms or for detecting plasmid-mediated AmpC beta-lactamases.

In the United States, many laboratories await NCCLS recommendations before attempting to detect new resistance mechanisms. Thus, many clinical laboratories attempt to detect ESBLs only in *E. coli* and *Klebsiella* spp. Some researchers suggest that this is the correct approach and that even discussion of such issues is unwarranted because it causes confusion. However, other organisms possessing these resistance mechanisms do cause infections, making this stance unacceptable. Moreover, the laboratory is an early warning system, alerting us to new resistance mechanisms in

Address for correspondence: Kenneth Thomson, Center for Research in Anti-Infectives and Biotechnology, Department of Medical Microbiology and Immunology, Creighton University School of Medicine, 2500 California Plaza, Omaha, NE 68178, USA; fax: 402-280-1225; e-mail: kstaac@creighton.edu patients. An early warning system that allows time lags of 12 or more years before new types of resistant organisms are detected is untenable. Twelve years is not an early warning, and laboratories that operate in this manner cannot meet their responsibility.

### NCCLS and the Emergence of New Pathogens

Can the current deficiencies be rectified? Two issues affect laboratories: the role of NCCLS and the speed with which new types of pathogens are emerging. NCCLS's task of creating laboratory test recommendations is difficult and often underappreciated. The committee has responsibilities in the areas of regulation, standardization, and safety. It is not NCCLS's role to be at the cutting edge of research, nor would it be appropriate for it, or other similar bodies, to be overly hasty and make decisions based on inadequate data. It can take years to gather data about a new, relatively uncommon, resistance mechanism. Time is also needed for analysis and debate. Properly done, the process cannot be rushed. The problem is that bacteria are evolving or adapting faster than this process.

Today, many bacterial pathogens are more complex than a decade or two ago. Thus, previously reliable susceptibility tests may no longer be dependable. For example, there are not only new resistance mechanisms, such as ESBLs, but also isolates that produce multiple beta-lactamases. Such organisms were not encountered often, if at all, when the current NCCLS susceptibility test criteria were prepared. For example, before the 1990s, *K. pneumoniae* isolates typically produced a single beta-lactamase, SHV-1, or occasionally two beta-lactamases (25-27). Today, *K. pneumoniae* isolates that produce three to six beta-lactamases are commonplace in some centers (28-34). Such changes necessitate new or modified tests to provide accurate and clinically relevant susceptibility reports. But instead of laboratory testing methods being upgraded during the last decade, the emphasis has been on cost-cutting and downsizing. Laboratories are under pressure to use cheaper, abbreviated tests or merely to maintain the technical status quo of a decade or more ago. In centers where the newer, more complex pathogens occur, reliance on the older tests leaves patients and institutions at risk.

### A More Responsive Approach

One approach to overcoming such problems would be to ensure that each laboratory has a staff member with the time, interest, and expertise to provide leadership in antibiotic testing and resistance. This person would read relevant publications, network with other laboratories, and evaluate potentially useful tests to detect new forms of resistance in the vulnerable interim period before new NCCLS-recommended tests become available. The person with this responsibility should work closely with reference laboratories, such as those of the Centers for Disease Control and Prevention or other sites with expertise. This would help to ensure that, whenever a new resistance mechanism is suspected, it would be properly checked, and the reference laboratory could provide feedback about whether the finding was "real."

### Unresolved Issues

The gaps in current laboratory knowledge and testing have generated several unresolved issues. One is whether positive, but unconfirmed, ESBL screens should be routinely reported. This is a consequence of the NCCLS two-step approach to ESBL detection. The first step is a screening for reduced susceptibility to any of the recommended screening agents (cefotaxime, ceftriaxone, ceftazidime, cefpodoxime, or aztreonam). Confirmatory testing, initiated only after a positive screening result, is based on tests with combinations of screening agents and the beta-lactamase inhibitor clavulanate. This testing indirectly detects hydrolysis of a screening agent by an ESBL by demonstrating potentiation of the activity of a screening agent in the presence of the beta-lactamase inhibitor. Confirmatory testing may require up to one extra day to detect ESBLs. If the laboratory reports a positive ESBL screening result to the physician and the isolate subsequently proves to be ESBL negative, the report could lead to unnecessary use of a carbapenem. Alternatively, if the laboratory withholds the positive screening result and the isolate is subsequently confirmed as ESBL positive, appropriate therapy may have been delayed for a day. Clearly, a reporting rule cannot cover all situations. Rather, the need to report a positive screening result should be determined on a case-by-case basis using common sense and experience as guides, taking into account the patient's status, infection control considerations, and the likelihood of a positive confirmatory test (based on prior experience with isolates from the same patient population). Using a reliable, rapid confirmatory test could minimize the time required for the second-step test and lessen this reporting dilemma. Another solution would be including ESBL confirmation testing in the routine susceptibility test.

Another issue is which NCCLS screening agent should be tested. Generally, the most reliable screening agent is the most sensitive. Cefpodoxime is the most sensitive ESBL screening agent for *K. pneumoniae* and *E. coli*, but a poor screening agent for *K. oxytoca* (35). The superior sensitivity of this agent can be accompanied by poor specificity in tests with some ESBL-negative *E. coli* isolates. This is another problem arising from the two-step approach to detecting ESBLs, which could be avoided by including a confirmatory test (ideally cefpodoxime plus clavulanate for *K. pneumoniae* and *E. coli* isolates) in the routine susceptibility test (17,36).

How best to detect ESBLs in organisms other than *Klebsiella* spp. *or E. coli* has not received much attention. The inhibitor-based confirmatory test approach is the most promising detection method (37). However, with isolates of some species, clavulanate is an unreliable agent for this test. The inhibitor-based approach is most reliable for isolates that do not coproduce an inhibitor-resistant beta-lactamase, such as AmpC. High-level expression of AmpC may prevent recognition of an ESBL. This problem is more common in tests with species or strains that produce a chromosomally encoded inducible AmpC beta-lactamase (e.g., *Enterobacter, Serratia*, *Providencia*, *Aeromonas* spp., *M. morganii*, *C. freundii*, *Hafnia alvei*, and *P. aeruginosa*). With these organisms, clavulanate may act as an inducer of high-level AmpC production and increase the resistance of the isolate to other screening drugs, producing a false-negative result in the ESBL detection test (Table 1). Tazobactam and sulbactam are much less likely to induce AmpC beta-lactamases and are therefore preferable inhibitors for ESBL detection tests with these organisms (37). Another possible solution is to include cefepime as an ESBL screening agent (38). High-level AmpC expression has minimal effect on the activity of cefepime, making this drug a more reliable detection agent for ESBLs in the presence of an AmpC beta-lactamase.

Table 1. Example of false-negative, clavulanate-based test for detecting extended-spectrum beta-lactamases with an isolate producing an inducible AmpC beta-lactamase[a]

| Isolate | Test agent | MIC (µg/mL) |
|---|---|---|
| SHV-2-producing *Enterobacter cloacae* | Ceftazidime alone | 2 |
| | Ceftazidime + 4 µg/mL clavulanate | 16 |

[a]Source: Thomson KS, Moland ES, Sanders CC (40).

A further concern with ESBL-producing organisms other than *Klebsiella* and *E. coli* is reporting their antibiotic susceptibilities. In Table 2, the beta-lactam MICs of an SHV-3-producing *C. freundii* isolate are within the NCCLS susceptible range of $\leq$8 µg/mL. If the isolate were *Klebsiella* or *E. coli*, the NCCLS reporting rule would apply, and the isolate would be reported as resistant to all penicillins, cephalosporins, and aztreonam. However, there is no ESBL reporting rule for other organisms; therefore, this organism would be reported as susceptible to cefotaxime, ceftazidime, aztreonam, and cefepime. This is inconsistent. Not only does this *C. freundii* isolate produce an ESBL, it also produces a chromosomal AmpC beta-lactamase that can hydrolyze the cephalosporins and aztreonam. It therefore seems wrong to report this organism as susceptible to these agents. Moreover, when the organism was tested at a 100-fold higher-than-standard inoculum, a dramatic inoculum effect occurred, with large increases in the MICs of these agents, analogous to the inoculum effect that occurs with ESBL-producing *Klebsiella* spp. and *E. coli* (Creighton University, unpub. data). This finding adds support for reporting all ESBL-producing isolates, not just *Klebsiella* spp. and *E. coli*, as resistant to all penicillins, cephalosporins, and aztreonam.

Detecting and reporting isolates producing plasmid-mediated AmpC beta-lactamases are more difficult issues than those associated with ESBLs. Detection is technically difficult in organisms that also produce a chromosomal AmpC, since proving that an AmpC is plasmid mediated, and not the usual chromosomal enzyme, is necessary. This determination is beyond the capabilities of most clinical laboratories. However, *Klebsiella* spp. do not possess a chromosomal AmpC. This makes them convenient indicator organisms to screen when attempting to detect plasmid-mediated AmpCs. Phenotypic tests for AmpC detection are not well defined. Screening tests could be based on decreased susceptibility to cephamycins. AmpC beta-lactamases are resistant to all marketed beta-lactamase inhibitors. Therefore, negative ESBL confirmatory tests based on these inhibitors may provide indirect evidence of AmpC production, or reduced outer membrane permeability. A positive three-dimensional test result with cefoxitin demonstrates hydrolysis of cefoxitin and differentiates between AmpC production and reduced outer membrane permeability (39). If an investigational AmpC beta-lactamase inhibitor were made available for diagnostic testing, it could be used in combination with a suitable cephem to confirm AmpC production.

Table 2. Standard and high-inoculum microdilution MICs in tests with SHV-3-producing *Citrobacter freundii* (MICs in µg/mL)[a]

| Inoculum (CFU/mL) | Cefotaxime | Ceftazidime | Aztreonam | Cefepime |
|---|---|---|---|---|
| 5 x $10^5$ | 2 | 1 | 0.5 | 0.5 |
| 5 x $10^7$ | 256 | 32 | 32 | >128 |

[a]Creighton University, unpub. data.

Susceptibility reporting may prove controversial for isolates producing plasmid-mediated AmpC beta-lactamases. Isolates that produce these enzymes can be susceptible in vitro to cephalosporins and aztreonam (Table 3). If these agents are used therapeutically for infections with such organisms, determining if they pose a treatment failure risk for patients is a priority.

Table 3. MICs associated with plasmid-mediated AmpC production in *Klebsiella pneumoniae* (MICs in µg/mL)[a]

| Enzyme | Cefotaxime | Ceftazidime | Aztreonam |
|---|---|---|---|
| FOX-1 | 4 | 8 | 0.5 |
| CMY-1 | 128 | 4 | 32 |

[a]Creighton University, unpub. data.

### Conclusions

Since clinical laboratories are first to encounter bacteria with new forms of antibiotic resistance, they need appropriate tools to recognize these bacteria, including trained staff with sufficient time and equipment to follow up important observations. Because bacterial pathogens are constantly changing, training must be an ongoing process. As we have learned from ESBLs, the methods and training that were previously adequate may no longer suffice against the newer types of pathogens. If laboratories continue to lag years behind new bacterial developments, new pathogens will spread, resulting in increasing problems and costs for patients and institutions.

Dr. Thomson is associate professor of medical microbiology and immunology and director of the Center for Research in Anti-Infectives and Biotechnology at Creighton University School of Medicine, Omaha, Nebraska. His major research and teaching interests are antibiotic resistance mechanisms, especially beta-lactamases of gram-negative bacteria, beta-lactam antibiotics, fluoroquinolone antibiotics, and the clinical relevance of antiobiotic susceptibility tests.

### References

1. Knothe H, Shah P, Krcmery V, Antal M, Mitsuhashi S. Transferable resistance to cefotaxime, cefoxitin, cefamandole and cefuroxime in clinical isolates of *Klebsiella pneumoniae* and *Serratia marcescens*. Infection 1983;11:315-7.
2. Bauernfeind A, Chong Y, Schweighart S. Extended broad-spectrum ß-lactamase in *Klebsiella pneumoniae* including resistance to cephamycins. Infection 1989;17:316-21.
3. Goussard S, Courvalin P. Updated sequence information for TEM beta-lactamase genes. Antimicrob Agents Chemother 1999;43:367-70.
4. Heritage J, M'Zali FH, Gascoyne-Binzi D, Hawkey PM. Evolution and spread of SHV extended-spectrum beta-lactamases in gram-negative bacteria. J Antimicrob Chemother 1999;44:309-18.
5. Jacoby GA, Medeiros AA. More extended-spectrum ß-lactamases. Antimicrob Agents Chemother 1991;35:1697-1704.
6. Marchandin H, Carriere C, Sirot D, Pierre HJ, Darbas H. TEM-24 produced by four different species of Enterobacteriaceae, including *Providencia rettgeri*, in a single patient. Antimicrob Agents Chemother 1999;43:2069-73.

7. Mugnier P, Dubrous P, Casin I, Arlet G, Collatz E. A TEM-derived extended-spectrum ß-lactamase in *Pseudomonas aeruginosa*. Antimicrob Agents Chemother 1996;40:2488-93.
8. Palzkill T, Thomson KS, Sanders CC, Moland ES, Huang W, Milligan TW. New variant of TEM-10 ß-lactamase gene produced by a clinical isolate of *Proteus mirabilis*. Antimicrob Agents Chemother 1995;39:1199-200.
9. Philippon A, Labia R, Jacoby GA. Extended-spectrum ß-lactamases. Antimicrob Agents Chemother 1989;33:1131-6.
10. Bauernfeind A, Chong Y, Lee K. Plasmid-encoded AmpC beta-lactamases: how far have we gone 10 years after the discovery? Yonsei Med J 1998;39:520-5.
11. Livermore DM. ß-lactamases in laboratory and clinical resistance. Clin Microbiol Rev 1995;8:557-84.
12. Philippon A, Arlet G, Lagrange PH. Origin and impact of plasmid-mediated extended-spectrum beta-lactamases. Eur J Clin Microbiol Infect Dis 1994;13(Suppl 1):S17-29.
13. Barnaud G, Arlet G, Verdet C, Gaillot O, Lagrange PH, Philippon A. *Salmonella enteritidis*: AmpC plasmid-mediated inducible ß-lactamase (DHA-1) with an ampR gene from *Morganella morganii*. Antimicrob Agents Chemother 1998;42:2352-8.
14. Tenover FC, Mohammed MJ, Gorton TS, Dembek ZF. Detection and reporting of organisms producing extended-spectrum beta-lactamases: survey of laboratories in Connecticut. J Clin Microbiol 1999;37:4065-70.
15. Paterson DL, Yu VL. Extended-spectrum beta-lactamases: a call for improved detection and control [editorial; comment]. Clin Infect Dis 1999;29:1419-22.
16. Babini GS, Livermore DM. Antimicrobial resistance amongst *Klebsiella* spp. collected from intensive care units in Southern and Western Europe in 1997-1998. J Antimicrob Chemother 2000;45:183-9.
17. Brun-Buisson C, Legrand P, Philippon A, Montravers F, Ansquer F, Duval J. Transferable enzymatic resistance to third-generation cephalosporins during nosocomial outbreak of multiresistant *Klebsiella pneumoniae*. Lancet 1987;ii:302-6.
18. Casellas JM, Goldberg M. Incidence of strains producing extended spectrum ß-lactamases in Argentina. Infection 1989;17:434-6.
19. Karas JA, Pillay DG, Muckart D, Sturm AW. Treatment failure due to extended spectrum ß-lactamase. J Antimicrob Chemother 1996;203-4.
20. Rice LB, Eckstein EC, DeVente J, Shlaes DM. Ceftazidime-resistant *Klebsiella pneumoniae* isolates recovered at the Cleveland Department of Veterans Affairs Medical Center. Clin Infect Dis 1996;23:118-24.
21. Venezia RA, Scarano FJ, Preston KE, Steele LM, Root TP, Limberger R, et al. Molecular epidemiology of an SHV-5 extended-spectrum beta-lactamase in Enterobacteriaceae isolated from infants in a neonatal intensive care unit. Clin Infect Dis 1995;21:915-23.
22. Paterson D, Ko W, Von Gottberg A, Mohapatra S, Casellas J, Mulazimoglu L, et al. In vitro susceptibility and clinical outcomes of bacteremia due to extended-spectrum ß-lactamase (ESBL)-producing *Klebsiella pneumoniae*. Clin Infect Dis 1998;27:956.
23. Thomson KS, Prevan PM, Sanders CC. Novel plasmid-mediated ß-lactamases in Enterobacteriaceae: emerging problems for new ß-lactam antibiotics. In: Remington JS, Swartz MN, editors. Current clinical topics in infectious diseases. Cambridge: Blackwell Science, Inc.; 1996. p. 151-63.
24. National Committee for Clinical Laboratory Standards. Performance standards for antimicrobial susceptibility testing; tenth informational supplement (aerobic dilution). Villanova (PA): National Committee for Clinical Laboratory Standards; 2000.
25. Sanders CC, Iaconis JP, Bodey GP, Samonis G. Resistance to ticarcillin-potassium clavulanate among clinical isolates of the family Enterobacteriaceae: role of PSE-1 ß-lactamase and high levels of TEM-1 and SHV-1 and problems with false susceptibility in disk diffusion tests. Antimicrob Agents Chemother 1988;32:1365-9.
26. Liu PY, Gur D, Hall LM, Livermore DM. Survey of the prevalence of beta-lactamases amongst 1000 gram-negative bacilli isolated consecutively at the Royal London Hospital. J Antimicrob Chemother 1992;30:429-47.
27. Reig R, Roy C, Hermida M, Teruel D, Coira A. A survey of beta-lactamases from 618 isolates of *Klebsiella* spp. J Antimicrob Chemother 1993;31:29-35.
28. Bradford PA, Urban C, Mariano N, Rahal J, Bush K. Imipenem (IPM) resistance in clinical isolates of *Klebsiella pneumoniae* (K.pn). Caused by ACT-1, a plasmid-mediated AmpC ß-lactamase combined with loss of an outer membrane porin protein. In: Program and Abstracts of the 36nd Interscience Conference on Antimicrobial Agents and Chemotherapy. Washington: American Society for Microbiology; 1996. p. 39.
29. Fournier B, Roy PH. Variability of chromosomally encoded beta-lactamases from *Klebsiella oxytoca*. Antimicrob Agents Chemother 1997;41:1641-8.
30. Gazouli M, Tzouvelekis LS, Prinarakis E, Miriagau V, Tzelepi E. Transferable cefoxitin resistance in enterobacteria from Greek hospitals and characterization of a plasmid-mediated group 1 ß-lactamase (LAT-2). Antimicrob Agents Chemother 1996;40:1736-40.
31. Hanson ND, Thomson KS, Moland ES, Sanders CC, Berthold G, Penn R. Molecular characterization of a multiply resistant *Klebsiella pneumoniae* encoding ESBLs and a plasmid-mediated AmpC. J Antimicrob Chemother 1999;44:377-80.
32. Papanicolaou GA, Medeiros AA, Jacoby GA. Novel plasmid-mediated beta-lactamase (MIR-1) conferring resistance to oxyimino- and alpha-methoxy beta-lactams in clinical isolates of *Klebsiella pneumoniae*. Antimicrob Agents Chemother 1990;34:2200-9.
33. Pörnull KJ, Rodrego G, Dornbusch K. Production of a plasmid-mediated AmpC-like ß-lactamase by a *Klebsiella pneumoniae* septicemia isolate. J Antimicrob Chemother 1994;34:943-54.
34. Winokur PL, Eidelstain MV, Stetsiouk O, Stratchounski L, Blahova J, Reshedko GK, et al. Russian *Klebsiella pneumoniae* isolates that express extended-spectrum ß-lactamases. Clin Microbiol Infect 2000;6:103-8.
35. Thomson KS, Sanders CC. A simple and reliable method to screen isolates of *Escherichia coli* and *Klebsiella pneumoniae* for the production of TEM- and SHV-derived extended-spectrum ß-lactamases. Clin Microbiol Infect 1997;3:549-54.
36. Jarlier V, Nicolas M-H, Fournier G, Philippon A. Extended broad-spectrum ß-lactamases conferring transferable resistance to newer ß-lactam agents in Enterobacteriaceae: hospital prevalence and susceptibility patterns. Rev Infect Dis 1988;10:867-78.
37. Thomson KS, Moland ES, Sanders CC. Use of microdilution panels with and without ß-lactamase inhibitors as a phenotypic test for ß-lactamase production among *Eschericia coli*, *Klebsiella* spp., *Enterobacter* spp., *Citrobacter freundii,* and *Serratia marcescens*. Antimicrob Agents Chemother 1999;43:1393-400.
38. Tzouvelekis LS, Vatopoulos AC, Katsanis G, Tzelepi E. Rare case of failure by an automated system to detect extended-spectrum beta-lactamase in a cephalosporin-resistant *Klebsiella pneumoniae* isolate [letter]. J Clin Microbiol 1999;37:2388.
39. Thomson KS, Sanders CC. Detection of extended-spectrum ß-lactamases in members of the family Enterobacteriaceae: Comparison of the double-disk and three-dimensional tests. Antimicrob Agents Chemother 1992;36:1877-82.
40. Thomson KS, Moland ES, Sanders CC. Use of microdilution panels with and without ß-lactamase inhibitors as a specific test for ß-lactamase production (ßL+) among *E. coli* and Klebsiella. In: Abstracts of the 37th Interscience Conference on Antimicrobial Agents and Chemotherapy. Washington: American Society for Microbiology; 1997. p. 86.
